# Effect of Dynamic Circuit Pressures Monitoring on the Lifespan of Extracorporeal Circuit and the Efficiency of Solute Removal During Continuous Renal Replacement Therapy

**DOI:** 10.3389/fmed.2021.621921

**Published:** 2021-09-23

**Authors:** Peiyun Li, Ling Zhang, Li Lin, Xin Tang, Mingjing Guan, Tiantian Wei, Lixin Chen

**Affiliations:** Department of Nephrology, West China Hospital of Sichuan University, Chengdu, China

**Keywords:** continuous renal replacement therapy, circuit pressures, extracorporeal circuit failure, access outflow dysfunction, solute removal efficiency

## Abstract

**Objective:** To observe the effects of dynamic pressure monitoring on the lifespan of the extracorporeal circuit and the efficiency of solute removal during continuous renal replacement therapy (CRRT).

**Materials and Methods:** A prospective observational study was performed at the West China Hospital of Sichuan University in the ICU. Analyses of the downloaded pressure data recorded by CRRT machines and the solute removal efficiencies, calculated by 2^*^Ce/(Cpre+Cpost), where Ce, Cpre, and Cpost are the concentrations of the effluent, pre-filter blood, and post-filter blood, respectively, were performed. Samples were collected at 0, 2, 6, 12, and 24 h when continuous veno-venous hemodiafiltration (CVVHDF) was used after the initiation of CRRT. Measurements in concentrations of creatinine, blood urea nitrogen, and β2-microglobulin in the plasma and effluent were recorded.

**Results:** Extracorporeal circuits characterized by moderate-to-severe (M–S) access outflow dysfunction (AOD) events, defined as access outflow pressure less than or equal to −200 mmHg for more than 5 min, had shorter median lifespans with no anticoagulation (32.3 vs. 10.90 h, *P* = 0.001) compared with the no M–S AOD events group. The significant outcome also existed in regional citrate anticoagulation (RCA) (72 vs. 42.47 h, *P* = 0.02). Moreover, Cox regression analysis revealed that the lack of M–S AOD events, RCA, or CVVHDF independently prolonged the circuit lifespan. All tested solutes removal efficiencies started to decline at 12 h. Furthermore, efficiencies of all solutes removal dropped obviously at 24 h when TMP ≥ 150 mmHg.

**Conclusion:** RCA and CVVHDF predicted a longer circuit lifespan. M–S AOD events were associated with a shorter circuit lifespan when RCA or no anticoagulant was used. Replacement of extracorporeal circuit could be considered when running time of filter lasted up to 24 h with TMP ≥ 150 mmHg.

## Introduction

Continuous renal replacement therapy (CRRT) slowly and effectively removes water and solutes from critically ill patients (1). Prolonging the lifespan of CRRT circuits is fundamental for better use of the extracorporeal circuit. The extracorporeal circuit, which is the key part of CRRT, consists of a vascular access outflow lumen, pre-filter tubing, a filter, post-filter tubing, an air-trap chamber, pre-vascular inflow tubing, and a vascular access inflow lumen. Frequent clotting in the extracorporeal circuit may lead to blood loss, shorter effective treatment times, and increased medical costs (2). Many factors might influence circuit survival, including anticoagulation, vascular access, CRRT treatment parameters (e.g., modality, filter membrane, blood flow rate), hematocrit, and blood coagulation (3–9). However, the mechanisms of extracorporeal circuit failure (ECF) are still not clear.

In the past, pressure data were obtained by manual recording every hour. With developments in science and technology, mainstream CRRT machines can continuously record changes in pressure, such as access outflow pressure (AOP), pre-filter pressure (PFP), effluent pressure (EP), and return inflow pressure (RIP), every minute during therapy and store the data on internal storage. A few trials have investigated the pressure changes during CRRT (10, 11), and stored pressures data can be downloaded into an Excel spreadsheet to obtain the detailed pressure data and the precise circuit lifespan (12).

Continuous renal replacement therapy removes waste and maintains the electrolyte and acid–base balance via various techniques, so it is logical to believe that the removal efficiencies of diverse sizes of solutes are different due to their distinct characteristics and removal methods. Previous studies that focused on solute removal predominantly focused on modality and pre-/post-dilution. Many influencing factors remain unknown. In addition, no trials have investigated the relationship between dynamic pressure monitoring and solute removal efficiency hindered by the extraction method. We hypothesized that continuous pressure changes during CRRT affect the extracorporeal circuit lifespan and solute removal efficiency.

## Materials and Methods

### Study Design

This prospective, observational, cohort study was performed in the ICU of the West China Hospital of Sichuan University, Chengdu China. The data were recorded from October 2018 to December 2019. The study was approved by the Institutional Review Board of West China Hospital, Sichuan University (2017-06). Informed consent was obtained from the patient or a responsible surrogate.

### Study Population

A total of 395 episodes of CRRT in 131 patients were included. These episodes represented 16,244.1 h of treatment. Eligibility criteria included patients age 18 years or greater who had received at least one episode of CRRT with CVVHDF or continuous veno-venous hemofiltration (CVVH) modality in the ICU. All circuits had been provided using a Prismaflex machine (Baxter, United States), because this device is the main equipment in hospitals for CRRT. Patients were excluded if using other blood purification therapies, such as plasma exchange. CRRT circuits that were side arms of an extracorporeal membrane oxygenation circuit were also excluded.

### CRRT Protocol

The choice of anticoagulant is determined by the clinical situation. RCA was the first option when there were no contraindications (e.g., severe acidosis, liver failure, severe hypoxemia) against the use of it in this center with low-molecular weight heparin (LMWH) or no anticoagulant as the alternative. Meanwhile, LMWH is preferred for anticoagulation in patients with existing diseases (such as thrombosis) needing heparin. No anticoagulant use should be considered in patients at high risk of bleeding and with contraindications of citrate. For all the patients, double-lumen venous catheters were used for vascular access. All femoral vascular access was achieved via 13-Fr dual-lumen catheters (Baxter, United States), and jugular access was achieved via 11.5-Fr catheters (Baxter, United States). The blood rate was maintained at 150–200 mL/min. CVVH was performed in the pre-dilution mode. CVVHDF was performed in the post-dilution mode, and the ratio of dialysate to replacement fluid was 1:1. The replacement fluid used was the standard bicarbonate-based solution (QINGSHAN LIKANG, China); details of the components are presented in the Supplementary Material. Extracorporeal circuit cessation occurred when the extracorporeal circuit clotted or clotting. Meanwhile, the circuit reaching the maximum recommended use (72 h) should also be changed. A total of 395 episodes of CRT in 131 patients were included. These episodes represented 16,244.1 h of treatment. Eligibility criteria included patients of age 18 years or greater who had received at least one machine-recorded episode of CRRT and used CVVHDF or CVVH. All circuits had been provided using a Prismaflex machine (Baxter, United States) with the AN69 ST150 filter (Baxter, United States). Patients were excluded if using other blood purification therapies, such as plasma exchange, or if CRRT circuits that were side arms of an extracorporeal membrane oxygenation circuit were used.

### Measurement of Pressure Dynamics in the Extracorporeal Circuit

The methods used to extract, store, and analyze the continuous pressure data were similar to those described in a previous publication (12). The pressure variables included minute access outflow pressure (AOP), effluent pressure (EP), pre-filter pressure (PFP), and return inflow pressure (RIP) from relevant circuit points. Transmembrane pressure (TMP), corresponding to the pressure of the filter membrane, was calculated from these data using the equation: TMP = (PFP + RIP)/2–EP. Access outflow dysfunction (AOD) was defined as an AOP −200 mmHg according to a previous study (10). We defined three types of AOD events on the basis of total minutes of AOD: mild (≤5 min), moderate (5 min < timing ≤ 60 min), and severe (time > 60 min).

### Sample Collection in the Extracorporeal Circuit During CRRT and Measurement

Samples (blood and effluent) were obtained at 2, 6, 12, and 24 h when CRRT was used in the post-dilution CVVHDF modality. The concentrations of blood urea nitrogen (BUN), creatinine (Cr), and β2-microglobulin in the plasma and effluent were measured in the clinical laboratory of West China Hospital of Sichuan University. Solute removal efficiency = 2^*^Ce/(Cpre+Cpost), where Ce, Cpre, and Cpost are the concentrations of the effluent, blood pre-filter, and post-filter, respectively. The data of solute removal efficiency were matched with the accurate pressures data at the same timepoint.

### Collection of Characteristics

Baseline patient demographics, including gender, age, diagnosis, weight, and height were established from existing hospital databases. Laboratory parameters and the sequential organ failure assessment (SOFA) score (13) before initiation of every episode of CRRT were conducted, including hemoglobin, platelets, prothrombin time (PT), indexed normalized ratio (INR), and activated partial thromboplastin time (APTT). If no blood test was realized at the start of the circuit, the closest blood test realized was considered. We collected the following CRRT treatment characteristics, including blood flow, dose, anticoagulation, modality, vascular access site, circuit survival, and the reason for extracorporeal circuit change as reported in the ICU charting system.

### Statistical Methods

Continuous variables were expressed as mean with standard deviation if normally distributed, or median with interquartile range (IQR) if non-normally distributed. Categorical variables are reported as count with percentage. Variability of pressures was defined as the standard deviation for all pressures. Comparisons of data from groups were analyzed using the one-way analysis of variance, Mann–Whitney-test, Chi-square test, or Fisher's test. Variables associated with extracorporeal circuit lifespan were analyzed using the Cox regression model. A *p*-value <0.05 was considered to be statistically significant. Data were analyzed using SPSS version 19.0 (SPSS Inc., Chicago, IL, United States). **Figures 3**, **4** were drawn by Graphpad prism version 7.0 (Graphpad, United States).

## Results

### Patients and Extracorporeal Circuits

**Figure 1:**
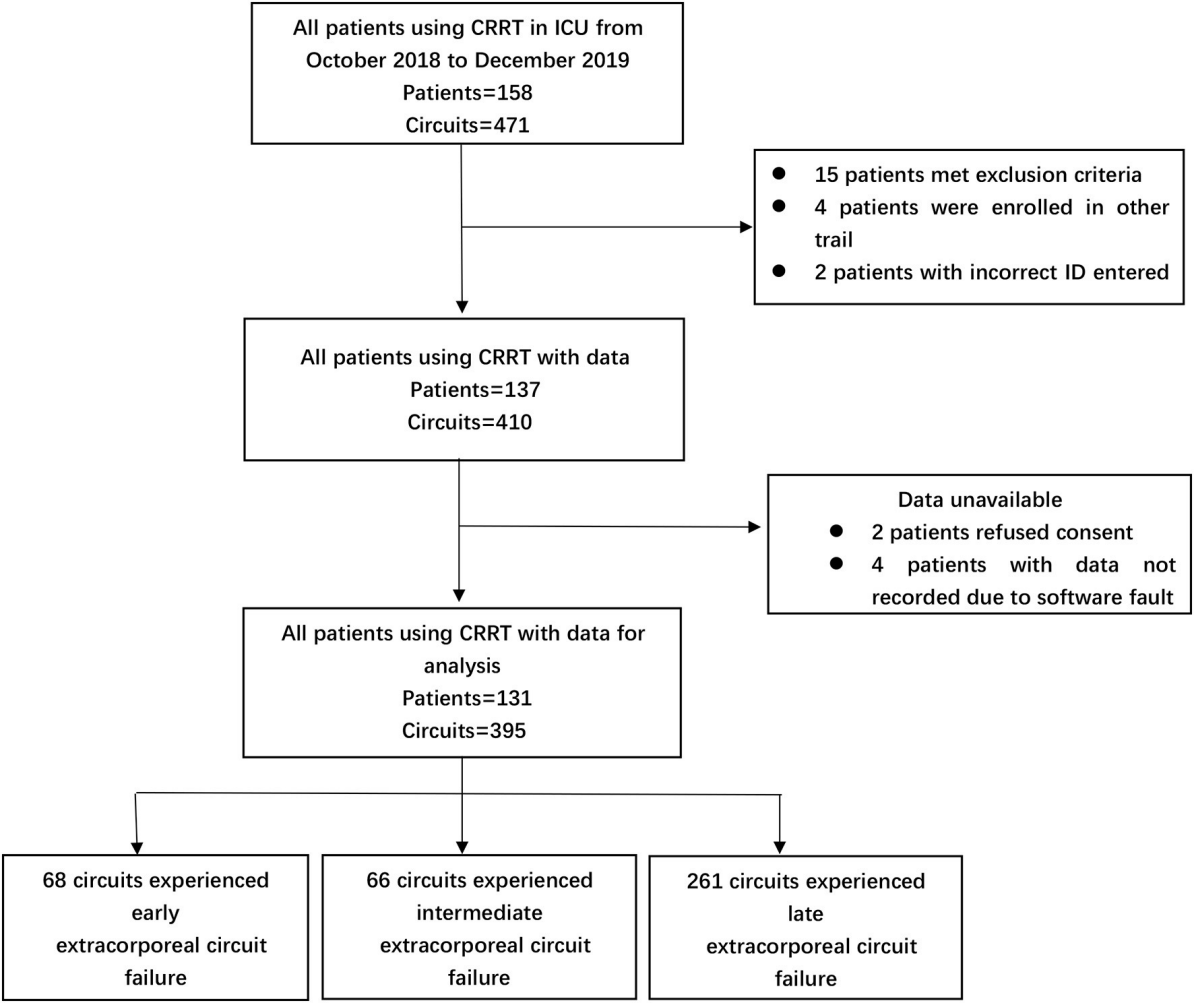
Numbers of CRRT episodes enrolled in the study, assigned to different extracorporeal circuit failures group.

A total of 395 episodes (Figure 1) in 131 patients, accounting for 16,244.1 h of effective treatment time, were included in the study. Over the course of our study, 96 cases (24.3%) were electively ended (i.e., the circuit had been used for 72 h). Clotting of the filter or air-trap chamber occurred in 299 cases (75.7%). The median lifespan of the extracorporeal circuit was 39.7 h. For anticoagulation, RAC was the primary choice (48.6%), followed by no anticoagulation (31.1%), and LMWH (20.3%). In the cluster of modality, the proportion of CVVHDF was 81.3%, and CVVH was 18.7%. The average prescribed dose of CRRT was 31.3 ± 3.2 ml/kg/h. The dominant access site was the femoral vein (368 circuits, 93.2%) with the remaining 27 circuits (6.8%) via a jugular vein. Two hundred twenty-seven circuits used the right side femoral vein as access and 141 circuits used the left side femoral vein. The details are reported in Tables 1a,b.

**Table 1a:** Demographics, clinical characteristics, and the extracorporeal circuit.

Age (years)	56.7 ± 14.0
Gender (male/female)	86/45
SOFA score	14 ± 2
*Diagnosis*	
Respiratory disease	33
Cardiovascular disease	30
Digestive diseases	43
Neurological disorders	5
Sepsis	20
Episodes of CRRT	395
*Reason for changing extracorporeal circuit*	
Clotting or clotted of extracorporeal circuit	299 (75.7%)
Elective circuit change	96 (24.3%)
*CRRT prescription*	
CVVH	74 (18.7%)
CVVHDF	321 (81.3%)
Dose of CRRT (ml/kg/h)	31.4 ± 3.1
Flow of blood (mL/min)	180.3 ± 10.1
*Laboratory data before the initiation of every episode of CRRT*	
Hemoglobin (g/L)	85.0 ± 19.0
Platelet (*10^9^/L)	113.0 ± 89.4
PT, s	18.0 ± 7.42
INR	1.5 ± 0.6
APTT, s	45.1 ± 21.7

*SOFA, the sequential organ failure assessment; CRRT, continuous renal replacement therapy; CVVH, continuous veno-venous hemofiltration; CVVHDF, continuous veno-venous hemodiafiltration*.

**Table 1b:** Lifespan of extracorporeal circuit in different groups.

**Variable**	**Circuits**	**Circuit life (h)**
All circuits	395	39.7 (6.91–72)
*Anticoagulation*		
Low molecular weight heparin	80 20.3%)	16.7 (8.5–33.0)
Regional citrate anticoagulation	192 (48.6%)	69.33 (37.29–72)
No anticoagulation	123 (31.3%)	29.42 (14.05–44.3)
*Vascular access*		
Internal jugular	27 (6.8%)	57.28 (33.73–72)
Right femoral	227 (57.4%)	33.43 (15.7–71.87)
Left femoral	141 (35.8%)	43.63 (18.55–72)
*Classification of extracorporeal circuit failure*		
Early	58 (14.7%)	7.0 (5.86–9.53)
Intermediate	76 (19.2%)	16.70 (13.97–20.60)
Late	261 (66.1%)	60.98 (39.78–72)

*First column expressed as absolute numbers, parentheses denote percentage of total. Second column expressed as median (interquartile range)*.

### Dynamic Pressure Changes During CRRT With Different Extracorporeal Circuit Failures

**Figure 2:**
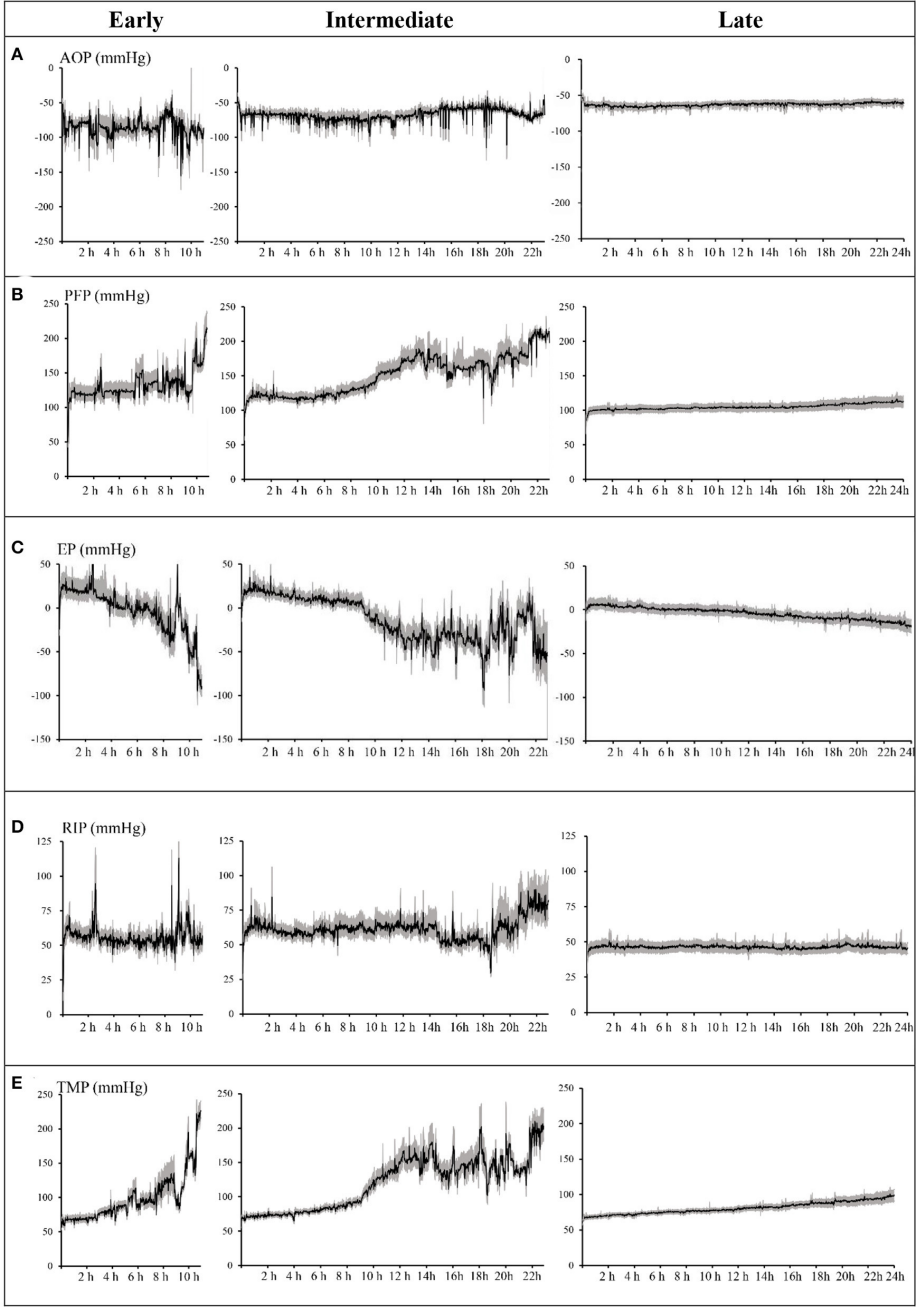
Dynamic mean pressure curve of every minute over time by early, intermediate, and late extracorporeal circuit failures. **(A)** Access outflow pressure (AOP). **(B)** Pre-filter pressure (PFP). **(C)** Effluent pressure (EP). **(D)** Return inflow pressure (RIP). **(E)** Transmembrane pressure (TMP). Shaded areas = 95% confidence of the mean. Lifespan of the early group ended at 11 h, the intermediate group ended at 23 h, and the late group ended at 24 h. AOP, PFP, EP, RIP, and TMP are the average values of each pressure minute.

For further analysis, according to the circuit lifespan we defined three types of ECFs (10), including early (≤12 h), intermediate (>12 h, ≤24 h), and late (>24 h). The median circuit life of these circuits was 7.0 h (IQR, 5.86–9.53 h) in the early ECFs group compared with 16.70 h (IQR, 13.97–20.60 h) in the intermediate group and 60.98 h (IQR, 39.78–72 h) in the late group. Overall, 134 circuits (33.9%) experienced early-intermediate failure, and 261 circuits (66.1%) experienced late failure. The mean changes in the AOP, PFP, EP, RIP, and TMP data were completely distinct in the different groups. The dynamic mean pressure curve graphs are shown in Figure 2. The negative value of AOP was smallest in the early group (−62.87 ± 2.31 mmHg), which was 23.5 and 4.87 mmHg lower than that in the late and intermediate groups, respectively. The overall changes in the PFP were also varied among the different types of ECFs: the mean value in the early, intermediate, and late groups were 133.43 ± 21.95, 150.47 ± 28.09, and 104.92 ± 3.89 mmHg, respectively. About EPs, the intermediate group had the smallest value of mean extracorporeal circuit data, followed by the late and early groups. In data of RIPs, the lowest and highest mean values were 46.38 ± 1.11 and 61.22 ± 7.74 mmHg in the late group and the intermediate group, respectively. In the curve graph of TMP, the line in the early and intermediate groups increased rapidly, with mean data of 98.12 ± 34.48 and 120.15 ± 38.891 mmHg, respectively. Moreover, the variability of the late groups was statistically smaller than that compared to the other groups (*P* < 0.05) in all totally different extracorporeal circuit pressure cluster (AOP, PFP, EP, RIP, TMP). The detailed variability data are shown in Table 2.

**Table 2:** Pressure data of different extracorporeal circuit failures.

**Pressure (mmHg)**	**Early ECF**	**Intermediate ECF**	**Late ECF**
AOP	−86.37 ± 13.03	−67.69 ± 8.20	−62.87 ± 2.31
AOP variability	37.73 ± 23.28	20.31 ± 14.33	13.79 ± 10.21
PFP	133.43 ± 21.95	150.47 ± 28.09	104.91 ± 3.89
PFP variability	32.86 ± 12.33	37.89 ± 15.20	12.67 ± 8.12
EP	−3.52 ± 26.07	−13.49 ± 25.03	−4.30 ± 6.44
EP variability	26.05 ± 13.73	37.04 ± 15.60	15.91 ± 9.81
RIP	55.82 ± 7.25	61.22 ± 7.74	46.37 ± 1.11
RIP variability	14.88 ± 8.39	15.53 ± 9.14	9.97 ± 5.99
TMP	98.12 ± 34.48	120.15 ± 38.89	80.79 ± 8.11
TMP variability	32.40 ± 16.12	46.40 ± 20.75	14.38 ± 11.64

*ECF, extracorporeal circuit failure; AOP, access outflow pressure; PFP, pre-filter pressure; EP, effluent pressure; RIP, pre-filter pressure; TMP, transmembrane pressure*.

### Access Outflow Dysfunction Events Under Different Anticoagulants

A total of 225 circuits experienced at least one AOD episode, and no significant difference was found (38.81 vs.40.38 h, *P* = 0.66) in the median lifespan of the circuits in which no AOD event occurred. However, the median circuits survival without M–S AOD events were associated with a longer circuit lifespan (42.50 vs. 17.14 h, *P* = 0.001). About anticoagulation, the median circuit survival for the filter using RCA was significantly longer compared with non-RCA [RCA (69.41 h: IQR, 37.29–72) vs. LWMH (16.7 h: IQR, 8.5–33.0) vs. none (29.42 h: IQR, 14.05–44.3, *P* < 0.05)]. Moreover, the effects of the interaction of anticoagulant and M–S AOD events on circuit survival were distinct. When no anticoagulant was used, the median lifespan of circuits without M–S AOD events was significantly prolonged compared with M–S occurred (32.3 h: IQR, 16.79–44.78 vs.10.90 h: IQR, 6.23–19.19, *P* = 0.001). The same effect existed while using RCA (72 h: IQR, 39.67–72 vs. 42.47 h: IQR, 19.79–68.13, *P* = 0.02). However, the effect of M–S AOD events on circuit survival disappeared with LMWH (11.80 h: IQR, 6.23–22.24 vs.11.27 h: IQR, 6.97–19.24, *P* = 0.61**)**.

### Risk Factors of Circuit Survival for First Circuit, Subsequent Circuits, and All Circuits

Comparison between the early-intermediate and the late groups revealed that M–S AOD episodes (22.4 vs. 8.0%, *P* < 0.001), platelet level (102.67 ± 90.11 vs. 133.46 ± 84.86 ^*^10^9^/l, *P* = 0.011), and CVVHDF modality (90.4 vs. 63.4%, *P* < 0.001) were different. However, mild AOD events, hemoglobin, PT, INR, APTT, and vascular access were not different between these two groups (Table 3). For the first circuit from each patient, there were 131 circuits for analysis. The study revealed that CRRT with the use of RCA was more likely to prolong circuit survival compared with use of no anticoagulant [HR, 0.44 (0.25–0.79), *P* = 0.006]. CVVHDF [HR, 0.38 (0.20–0.74), *P* = 0.004] was associated with longer circuit lifespan. Meanwhile, M–S AOD event [HR, 3.80 (1.50–9.62), *P* = 0.005] was highly in connection with ECF. Excluding the first filter, for subsequent circuits to analyze, no M-S AOD event, RCA, and CVVHDF were still intensively associated with longer lifespan of extracorporeal circuit. However, higher hemoglobin was slightly associated with longer circuit survival [HR, 0.91 (0.84–0.97), *P* = 0.006]. The analysis involved all circuits that showed no M–S AOD event, RAC, CVVHDF, lower platelets levels, higher hemoglobin were independently associated with longer circuit lifespan (detailed data shown in Table 4). In summary, no M–S AOD even t, RAC, and CVVHDF remained associated with greater circuit survival.

**Table 3:** Comparisons between the early-to-intermediate and late ECF groups.

	**Early-to-intermediate ECF, *N* = 134**	**Late ECF, *N* = 261**	***P*-value**
With AOD	83 (61.9%)	142 (54.4%)	0.22
With mild AOD	54 (40.3%)	121 (46.4%)	0.34
With M-S AOD	30 (22.4%)	21 (8.0%)	<0.001
Hemoglobin	82.67 ± 18.04	86.23 ± 19.40	0.17
Platelet (*10^9^/L)	133.46 ± 84.86	102.67 ± 90.11	0.01
PT, s	16.93 ± 6.07	17.85 ± 14.42	0.58
INR	1.54 ± 0.54	1.51 ± 0.74	0.73
APTT, s	41.48 ± 19.26	45.55 ± 24.40	0.20
Modality (CVVHDF)	85 (63.4%)	236 (90.4%)	<0.001
Vascular access (femoral)	129 (96.3%)	239 (91.6%)	0.14
Location of femoral vein (right)	79 (61.2%)	132 (55.4%)	0.38

*AOD, access outflow dysfunction; M-S AOD, moderate-severe access outflow dysfunction; PT, prothrombin time; INR, international normalized index; APTT, activated partial thromboplastin time; CVVHDF, continuous veno-venous hemodiafiltration*.

**Table 4:** Cox regression analysis of variables associated with shorter circuit survival.

**Variables**	**First circuit**		**Subsequent circuits**		**All circuits**	
	**HR (95%CI)**	***P*-value**	**HR (95%CI)**	***P*-value**	**HR (95%CI)**	***P*-value**
AOD	0.92 (0.53–1.61)	0.76	0.99 (0.71–1.40)	0.96	0.95 (0.72–1.27)	0.75
Moderate-severe AOD	3.79 (1.5–9.62)	0.005	1.66 (1.10–2.55)	0.02	1.88 (1.28–2.74)	0.001
*Anticoagulation (relative to none)*						
Regional citrate anticoagulation	0.44 (0.25–0.79)	0.006	0.41 (0.27–0.62)	<0.001	0.42 (0.30–0.58)	<0.001
LWMH	1.35 (0.68–2.67)	0.39	1.34 (0.86–2.08)	0.20	1.30 (0.90–1.88)	0.17
CVVHDF	0.38 (0.20–0.74)	0.004	0.47 (0.29–0.74)	0.001	050 (0.34–0.72)	<0.001
Platelets (per 100 G/L increase)	1.09 (0.80–1.50)	0.58	1.11 (0.97–1.28)	0.13	1.13 (1.0–1.28)	0.048
Hemoglobin (per 10 g/L increase)	0.99 (0.90–1.08)	0.75	0.91 (0.84–0.97)	0.006	0.94 (0.90–0.99)	0.02

*AOD, access outflow dysfunction; LWMH, Low molecular weight heparin; CVVHDF, continuous veno-venous hemodiafiltration; HR, hazards ratio*.

### Solute Removal Efficiency and Dynamic Pressure Changes

**Figure 3:**
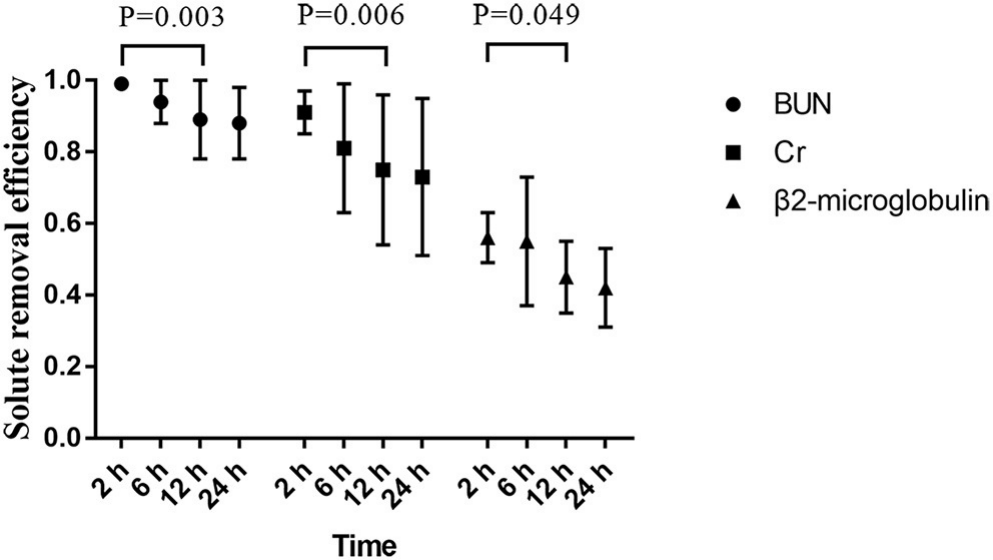
Solute removal efficiency at different times.

**Figure 4:**
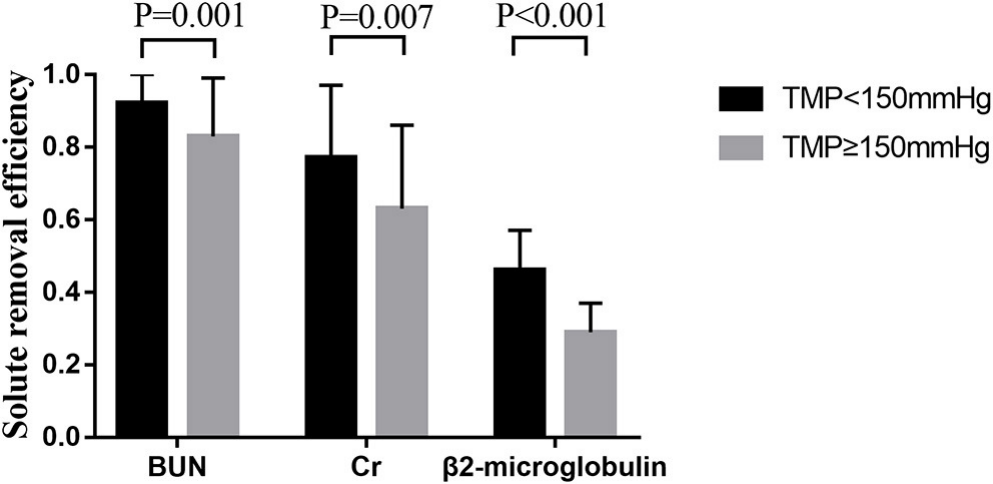
Solute removal efficiency in different TMPs groups at 24 h.

The removal efficiency of middle-molecular solute (β_2_-microglobulin) was significantly lower than that of BUN and creatinine at different time points during CRRT. All efficiencies of tested solutes removal (BUN, creatinine, and β2-microglobulin) dropped gradually with operation time prolonged (Figure 3). According to the precise TMP data which was matched with sample collection time, groups of TMP data were clustered into four (TMP <100 mmHg, 100 ≤ TMP <150 mmHg, 150 ≤ TMP <200 mmHg, TMP ≥ 200 mmHg), details presented in the Supplementary Material. In the comparison of different TMPs, two groups were formed: TMP <150 mmHg and TMP ≥ 150 mmHg. The solute removal efficiency in the lower TMP group showed a greater clearance ability than that in the higher TMP group. Moreover, this phenomenon significantly occurred between the TMP <150 mmHg and TMP ≥ 150 mmHg group for BUN (0.92 ± 0.10 vs. 0.83 ± 0.16, *P* = 0.001), creatinine (0.77 ± 0.20 vs. 0.63 ± 0.23, *P* = 0.007), and β2-microglobulin (0.46 ± 0.11 vs. 0.29 ± 0.08, *P* < 0.001) at 24 h (Figure 4).

## Discussion

### Main Findings

We analyzed continuous pressure data from CRRT and found that, after classifying the different types of circuit failures, M–S AOD was associated with a shorter lifespan of extracorporeal circuit compared to mild dysfunction. Moreover, when anticoagulation was performed with citrate or when anticoagulation was not performed, M–S was associated with shorter circuit survival compared to that observed when LMWH was used. We found that the use of CVVHDF and citrate and the absence of M–S AOD events prolonged the lifespan of the extracorporeal circuit. Our study demonstrated a distinct downtrend in small-molecule and middle-molecular solutes in removal efficiency under different anticoagulation modalities. Solutes removal efficiency declined significantly at 24 h or TMP ≥ 150 mmHg. Meanwhile, removal efficiency declined when circuit survival up to 24 h while TMP ≥ 150 mmHg compared with those in TMP <150 mmHg at 24 h.

### Relationship to Previous Studies

#### Lifespan of Extracorporeal Circuit

Recently published studies (3, 4) suggested that RCA was superior to heparin for circuit survival and anticoagulation-related bleeding risk. However, the lifespan of the extracorporeal circuit still varied greatly in studies despite whatever anticoagulant was applied. A multicenter, randomized controlled study (14) of 174 patients compared circuit survival when different anticoagulants were used, namely, citrate and heparin, during CRRT. The lifespan of the two groups was 37.5 ± 23 h and 26.1 ± 19 h, respectively. The standard deviation confirmed the variability in circuit survival. Brain et al. (9) reported a meta-analysis about non-anticoagulant factors (such as vascular access, dialysis membrane, and modality) on the lifespan of the extracorporeal circuit, but the value of this article decreased because most of the studies were observational or reported circuit factors in sub-analysis. Factors influencing the lifespan of the extracorporeal circuit are not exactly definite, so further studies are needed. AOP is a major concern in circuit pressures monitoring on the lifespan of the extracorporeal circuit. AOP is measured between the catheter and the blood pump. Since the inner blood is sucked by the extracorporeal circuit, the AOP is generally negative and < −50 mmHg (15). A recently published observational study (10) was the first study to acquire continuous pressure data accurately during CRRT, and these pressures accurately reflect the real state of each part of the extracorporeal circuit. This study suggested that an AOP ≤−200 mmHg could be considered a dysfunction, and AOD events can shorten the survival of the extracorporeal circuit. The study still had some limitations, such as the inclusion of a narrow population (most were post-operative patients) and the lack of RCA data. A recent retrospective study (11) suggested that the occurrence of an AOD event within 4 h after the initiation of CRRT significantly reduced the lifespan of the extracorporeal circuit by 12.9 h compared with the absence of an AOD event. COX analysis of two studies (10, 11) suggested that AOD events were independent risk factors for circuit survival, which indicates that AOP status warrants concern.

AOD events are quite common in the clinic, and these events are an indirect indicator of the quality and function of the vascular access. Several causes of AOD were proposed: 1. The patient's body position may change frequently due to the needs of nursing or other therapy. The catheter may be suddenly bent or folded, which results in a sharp decrease in AOP and an extremely negative value. This interference is the most common reason for an AOD event in the clinic (16, 17). 2. The formation of thrombus or fibrous sheath in the lumen of a catheter or the collapse or thrombosis of the central vein where the catheter was placed may cause an AOD event. 3. Blood flow exceeding the maximum allowable range of the double-lumen catheter (>350 or 400 ml/min) may also cause an AOD event. The occurrence of M–S AOD events should be avoided as much as possible. The results of our study suggested that short-term AOD is not enough to affect the lifespan of the extracorporeal circuit. Only AOD that lasted a sustainable time (≥5 min), such as an M–S AOD event, affected the extracorporeal circuit, especially circuits with citrate and no anticoagulation. Notably, this phenomenon did not indicate that heparin were superior to RCA and no anticoagulation but only indicated that M–S AOD events should be a concern. The possible explanation for this result is that different anticoagulants play distinct roles. Citrate prevents coagulation by complexing ionized calcium in the extracorporeal circuit. The part entering the human body is metabolized from one molecule of citrate into three molecules of bicarbonate in the mitochondria of the liver, skeletal muscle, and kidney (18). Notably, complexed calcium is released, and lost calcium is supplemented in post-filter. Therefore, citrate is an ideal regional anticoagulant that effectively maintains an anticoagulation effect in the extracorporeal circuit and avoids bleeding in the body. LMWH exerts systemic anticoagulant effects by enhancing antithrombin III activity and inhibiting thrombin (factor IIa) and factor Xa. The pharmacokinetics are complex. Therefore, the variability in the high risk of bleeding individuals is a disadvantage. In addition, COX analysis showed that M–S AOD events were a risk factor for circuit survival.

#### Solute Removal Efficiency

The use of RCA has been verified to prolong the circuit survival and avoid a system “shutdown” because of the early clotting of the circuit. Nevertheless, a decrease in solute clearance occurs even if the extracorporeal circuit functions properly. From now on when we should replace the extracorporeal circuit accurately is a mystery and even the Kidney Disease: Improving Global Outcomes (KDIGO) guidelines do not have a suggestion about that point, and how and when do solute removal efficiency decay are still indeterminate. Therefore, it is very valuable to find an indicator to determine whether to replace the extracorporeal circuit. Clogging of hemofilter membranes and clotting of the circuit are associated with the rise in TMP (15). Compared with other pressures data, TMP is particularly important in the study of solute removal efficiency. The relationship between TMPs and solute removal efficiency has not been investigated. Previous trials have studied the effects of diverse filter membranes and dilution methods on removal efficiency (19, 20). A large multicenter randomized controlled (RENAL) study (21) of 1,508 patients investigated the effect of high dose (40 ml/kg^*^h) and low dose (25 ml/kg^*^h) on 90-day survival rate during CRRT and suggested no difference. A uniform CRRT dose was used in our study to exclude its effect on solute removal efficiency. A study (19) focused on the effect of membrane materials (Sureflux150E vs. AV-400) on solute clearance; however, the results showed no difference between cellulose triacetate membranes and synthetic membranes on the removal of solutes (urea nitrogen and creatinine). Our study only used ST150 membrane (polyacrylonitrile material) to decrease the interference of materials. A small multicenter randomized controlled study (22) recently focused on the effects of different modalities (CVVH *vs*. CVVHD), convection and diffusion, on solute clearance using similar doses. The results showed no significant difference at 0 h and 4 h (*P* > 0.05) for small solutes (urea nitrogen and creatinine) and medium-to-macromolecules (inflammatory mediators, such as IL-6). No study has analyzed the solute removal efficiency and continuous pressure in the extracorporeal circuit because of the prior lack of effective data extracting methods. Therefore, our study is innovative.

Solutes have distinct removal efficiencies due to unique characteristics. The kidney is the only excretory organ of β2-microglobulin (11.8 kDa). A previous study (23) showed that the risk of death increased 11% when the concentration of β2-microglobulin increased by 10 mg/L in blood. Therefore, this study selected it as a representative medium-molecular solute. It has been thought that small molecules, such as urea nitrogen, freely pass through the dialysis membrane for 100% removal. However, a randomized controlled study conducted by Lyndon et al. (24) revealed that the measured clearance rates of urea nitrogen and creatinine in a high-dose group during CRRT were significantly different from the achieved clearance rates of 7.1 and 13.9% (*P* < 0.001), respectively. The results showed that the clearance of urea nitrogen and creatinine was not 100%, and the ability to remove creatinine was significantly overestimated compared with urea nitrogen. However, this study had some limitations, such as the lack of a downward trend in the removal effects for diverse solutes. A recent prospective cohort study (25) investigated the effect of high-flux filters (surface area 1.8 m^2^) on the clearance of various solutes during CRRT. The results showed that the clearance of small molecule solutes (Cr and BUN) was not different at 72 h (0.99 ± 0.03 vs. 0.91 ± 0.16, *P* = 0.074; 1 ± 0 vs. 0.95 ± 0.17, *P* = 0.5), but β2-microglobulin changed substantially (0.61 ± 0.09 vs. 0.48 ± 0.13, *P* = 0.002). The results of this study are higher than our results at every sample collection time. The explanation for this phenomenon may be that the removal efficiency of the high-flux filter was higher than an ordinary filter. In addition, the lifespan of all the circuits was extreme (72 h), and no filter coagulation occurred with the use of citrate as the anticoagulation. Therefore, solute removal may decrease more slowly when the extracorporeal circuit is running well.

### Strengths and Limitations

This study has important clinical significance because continuous pressures data are still not completely utilized. In our study, we collected various modalities of anticoagulation and multiple RCA data (48.6%) compared to other trials (10, 11). Moreover, we creatively combined the dynamic pressure monitoring with the solute removal efficiency during CRRT and offered a new idea for circuit replacement.

Our study also has several limitations. First, it was a single-center observational study, so that its discoveries do not demonstrate causality. Also, the findings require verification by larger multicenter studies. In addition, our study used data from a single type of machine, dose, dialyzer membrane, so risk factors of circuit survival and the results of AOD need more various data to confirm the results. Besides, data lacked adjusting for the effect of within-patients repeated measurements. Finally, this study was short of data about solute removal efficiency of other middle molecular weight molecules (e.g., cytokines), so further study should be undertaken to corroborate these findings.

## Conclusion

RCA and CVVHDF prolonged circuit survival during CRRT. M–S AOD events should be of a concern, especially when RCA or no anticoagulant is used. With the prolonged use of the extracorporeal circuit, all tested solutes removal efficiency started to significantly decline at 12 h. Besides, with the increase of TMP, solute removal efficiency descended dramatically. Moreover, extracorporeal circuit might consider to be replaced at 24 h when TMP ≥ 150 mmHg because of the decline of solute removal efficiency.

## Data Availability Statement

The original contributions presented in the study are included in the article/Supplementary Material, further inquiries can be directed to the corresponding author.

## Ethics Statement

The studies involving human participants were reviewed and approved by Institutional Review Board of West China Hospital, Sichuan University. The patients/participants provided their written informed consent to participate in this study.

## Author Contributions

LZ mainly responsible for program design and modification. PL, LL, XT, MG, TW, and LC were involved in this clinical trial and vouch for the adherence of the trial to the protocol, for the accuracy of the data. PL conducted the statistical analysis and wrote the first draft. All authors reviewed, revised, and approved the final version of the manuscript and agreed to the submission of this paper.

## Funding

This study was funded by 1·3·5 project for disciplines of excellence–Clinical Research Incubation Project, West China Hospital, Sichuan University (18HXFH018).

## Conflict of Interest

The authors declare that the research was conducted in the absence of any commercial or financial relationships that could be construed as a potential conflict of interest.

## Publisher's Note

All claims expressed in this article are solely those of the authors and do not necessarily represent those of their affiliated organizations, or those of the publisher, the editors and the reviewers. Any product that may be evaluated in this article, or claim that may be made by its manufacturer, is not guaranteed or endorsed by the publisher.
