# Supplementary material for: Effect of Dynamic Circuit Pressures Monitoring on the Lifespan of Extracorporeal Circuit and the Efficiency of Solute Removal During Continuous Renal Replacement Therapy
Source: Front Med (Lausanne). 2021 Sep 23;8:621921. doi: 10.3389/fmed.2021.621921 (PMC8494973; doi:10.3389/fmed.2021.621921)
Supplement: Supplementary file 1 [file Table_1.DOCX]

**e-table1 The specific composition of the replacement fluid**

|  | **specific components** |
| --- | --- |
| Solution A | solution A (Glucose 10.6 mmol/L, Cl- 118 mmol/L, Mg2+ 0.797 mmol/L, Ca2+ 1.60 mmol/L, and Na+ 113 mmol/L) |
| Solution B | solution B (5% sodium bicarbonate). |

**e-table2 Solute removal efficiency in different TMPs groups**

| **Variables of**  **Solute removal efficiency** |  | **TMP<100 mmHg** | **100 ≤TMP<150 mmHg** | **150≤TMP<200 mmHg** | **TMP≥200 mmHg** |
| --- | --- | --- | --- | --- | --- |
| blood urea nitrogen |  | 0.94±0.03 | 0.87±0.12***** | 0.81±0.12 | 0.77±0.18 |
| creatinine |  | 0.80±0.18 | 0.70±0.23***** | 0.65±0.25 | 0.61±0.22 |
| β2-microglobulin |  | 0.48±0.12 | 0.41±0.08***** | 0.27±0.13 | 0.30±0.02 |

* suggested that there was statistical difference compared with TMP<100 mmHg group.

TMP: transmembrane pressure.
